# Instrument for classifying dependency in neonatal intensive care: analysis of agreement and reliability*

**DOI:** 10.1590/1518-8345.6922.4272

**Published:** 2024-08-30

**Authors:** Paula Mara Bordim Lopes, Fernanda Broering Gomes Torres, Thomaz Jefferson Massaneiro, Adriano Akira Hino, Elenice Valentim Carmona, Marcia Regina Cubas

**Affiliations:** 1 Pontifícia Universidade Católica do Paraná, Curitiba, Paraná, PR, Brazil.; 2 Pontifícia Universidade Católica do Paraná, Escola de Medicina, Curitiba, Paraná, PR, Brazil.; 3 Universidade Estadual de Campinas, Faculdade de Enfermagem, Campinas, São Paulo, SP, Brazil.

**Keywords:** Health Assessment, Neonatal Nursing, Validation Studies, Neonatal Intensive Care Units, Nursing Care, Patient Care Planning

## Abstract

**Objective:**

to analyze the reliability of the items that compose the instrument for classifying newborns according to the degree of dependence on nursing care in a neonatal intensive care unit.

**Method:**

methodological study that analyzed the agreement and reliability of the instrument in a neonatal intensive care unit. Six care nurses and a research nurse assessed 35 newborns and completed the instrument, which was made up of 15 areas of care. The weighted Kappa coefficient and the Intraclass Correlation Coefficient were used for analysis.

**Results:**

the areas of: weight (92%), oxygenation (93%) and catheter control (95%) had almost perfect agreement and the area of reaction to stimuli (50%) had poor agreement. The areas of elimination and vital signs showed low reliability, due to the low variability of responses. The Intraclass Correlation Coefficient was 0.94.

**Conclusion:**

there are variations in the evaluations of some areas of care due to the imprecise description of items to which scores are assigned, however the instrument is reliable for categorizing the type of care (minimal, intermediate and intensive). Its use can contribute to measuring the quality and safety of newborn care.

## Highlights

**(1)** The instrument is reliable for categorizing the type of newborn care.

**(2)** The use of a manual can minimize the subjectivity of the instrument.

**(3)** There is almost perfect agreement in less subjective areas of the instrument.

## Introduction

Every year, more than 15 million premature babies are born in the world^1^, prematurity is considered the main cause of admissions to the Neonatal Intensive Care Unit (NICU). In addition to prematurity and its complications, other clinical situations in the neonatal period require intensive care. 

Thus, the NICU is intended for newborn babies (NB) who require complex care of varying degrees and its purpose is to provide health care with technological resources, quality professionals and safety^2^. Neonatology nurses need to identify the specific needs of each NB in order to plan and organize safe, quality nursing care. In this sense, some tools can support neonatal nurses in identifying the results of actions in clinical practice^3^, in assessing the complexity of cognitive nursing workload^4^ *,* in measuring the intensity of treatments and for sizing the nursing team^5^.

With the aim of classifying NBs according to their degree of dependence on nursing care, an instrument was developed in 2000 for profiling NBs in the NICU^6^. Initially, the tool covered 16 areas of care: thermoregulation, weight, spontaneous activity, reaction to stimuli, skin color, muscle tone, nutrition and hydration, oxygenation, mucosal skin integrity, temperature, respiratory rate, heart rate, blood pressure, oxygen saturation, control of drains, probes and catheters, and drug therapy.

In 2005, the instrument was restructured for the Brazilian context to adapt it to individualized care needs, and the nutrition and hydration area was removed. As a result, the version intended for use in Brazil consists of 15 areas of care^6^, which is the version used in this study.

Although the newborn assessment and classification tool has undergone a content validation process, no study has been identified that has investigated its reliability in clinical practice, which is encouraged by the tool’s authors^6^. Analyzing the reliability of instruments implemented in care practice is fundamental to ensuring the safety of the instrument, proposing adjustments and sizing up nursing care for newborns^3^ ^)-(^ ^5^.

Thus, in order to contribute to the process of validating the instrument, the aim of this research was to analyze the reliability of the items that make up the instrument for classifying NB according to the degree of dependence on nursing care in a NICU.

## Method

### Type of study

This is a methodological study that analyzed the agreement and reliability of an instrument for classifying newborns according to their degree of dependence on nursing care in a neonatal intensive care unit.

### Setting of data collection

The study was carried out in the NICU of a private hospital in the city of Curitiba, Paraná, Brazil. In this hospital, the instrument for classifying NBs according to their degree of dependence on nursing care^6^ is used in routine care, having been incorporated into the electronic patient record system in 2017.

### Period

Data collection for this study took place between December 2021 and February 2022.

### Population

The study included six NICU nurses and 35 NBs admitted to the sector during the collection period. In the hospital’s NICU, the main reasons for admitting NBs were prematurity and/or respiratory distress.

### Selection criteria

All the nurses in the sector were selected to take part in the study, with the inclusion criterion being a specialist in neonatology, while the exclusion criterion was being on vacation or on leave during the data collection period. All NBs admitted to the department during the data collection period, whose parents agreed to take part in the study, were included in the study, with no exclusion criteria. 

### Sample definition

The sample was probabilistic and systematic, with a minimum proportion of 210 evaluations and a maximum of 420 evaluations of all participating NBs. 224 evaluations were carried out. The average number of assessments per NB was eight consecutive days.

### Study variables

The study variables comprise the areas of the NB classification tool^6^, they are:

- Thermoregulation - the ability to maintain a stable body temperature, with minimal caloric expenditure and oxygen consumption, for successful extrauterine adaptation;
- Weight - the need to monitor weight in order to compare daily weight with birth weight and with the previous day’s weight, helping to assess nutritional conditions and potential risks arising from birth weight;
- Spontaneous activity - ability to maintain a state of consciousness, behavioral response to sensory, proprioceptive, biochemical, thermal and mechanical stimuli and adequate physiological parameters for successful extrauterine adaptation;
- Reaction to stimuli - ability to respond to sensory, proprioceptive, biochemical, thermal and mechanical stimuli suitable for successful extrauterine adaptation;
- Skin color - ability to maintain adequate skin and mucous membrane color for successful extrauterine adaptation;
- Tonicity - ability to maintain vigorous muscle tone suitable for successful extrauterine adaptation;
- Elimination - ability to maintain spontaneous urinary and intestinal elimination with the help of others or through drains and stoma;
- Oxygenation - ability to maintain airway patency and gas exchange balance by oneself or with the help of nursing staff and/or equipment;
- Skin and mucous membrane integrity - ability to maintain skin and mucous membranes without damage or destruction;
- Body care - ability to maintain personal hygiene, clothing;
- Control of vital signs (CVV) - need to observe and control vital parameters - temperature (T), respiratory rate (RR), heart rate (HR), blood pressure (BP), O2 saturation;
- Control of probes and drains - need to observe and control equipment containing infusion and/or drainage fluids;
- Control of venous catheters - need to observe and control infusion and/or collection catheters, hemodynamic monitoring and hypertonic parenteral nutrition;
- Drug therapy - use of the various therapeutic medications - drugs, solutions, blood and blood products;
- Health education - the ability, confidence and security of the mother/family to provide adequate care to maintain the NBs’ personal and/or environmental health habits.

### Instruments used to collect information

The instrument used to assess and classify NB^6^ is made up of 15 areas of care, with one question for each area and a weight of one to three assigned to each item. Weight one corresponds to the lowest degree of dependence on nursing care and weight three to the highest degree^6^. For the purposes of analyzing the overall nursing care dependency score, all the answers are added together, resulting in a minimum score of 15 and a maximum of 45 points. The sum of the points corresponds to three categories of care: minimal (15 to 25 points), intermediate (26 to 36 points) and intensive (37 to 45 points). In order to standardize the scores, the authors of the instrument defined the meaning of each category and assigned the distribution of the score, taking into account the clinical instability of the NB and the answers offered by the judges, after applying the Delphi technique, with an agreement rate of over 70%^6^.

There was communication between the main author of the classification tool^6^ and the first author of this manuscript to present the aim of the research and provide clarification. Because the instrument was published in full, there was no formal authorization, only acknowledgement of use.

### Data collection

The 35 NBs were assessed daily, twice and at the same time by the research nurse and a care nurse, with the instrument being filled in at the same time as the assessment. The researcher did not interfere in the nurse’s assessment. These strategies were used to minimize data collection between evaluators.

### Data processing and analysis

In order to describe the 15 items that cover the areas of care, the absolute and relative frequency distribution of each area of care in which there was agreement between the research nurse and the care nurse was calculated. The same was done for the total number of concordant cases (n and % agreement). 

To assess the reliability of each item in the instrument, the weighted Kappa coefficient (Kp) was used, considering that the answer options were not dichotomous^7^. Kp ranges from 0 to 1, and the closer it is to 1, the greater the agreement between the evaluators. To interpret the Kp value, the corresponding intervals were classified as follows: less than 0.00 - insignificant agreement; from 0.00 to 0.20 - weak agreement; from 0.21 to 0.40 - reasonable agreement; from 0.41 to 0.60 - moderate agreement; from 0.61 to 0.80 - strong agreement; and from 0.81 to 1.00 - almost perfect agreement^8^.

The Intraclass Correlation Coefficient (ICC) was used to assess the reliability of the overall NB care dependency score generated by the sum of all the answers. In order to interpret it, the following classification was used: less than 0.5 - low reliability; from 0.5 to 0.75 - moderate reliability; from 0.75 to 0.90 - good reliability; and greater than 0.90 - excellent reliability^9^. 

Finally, in order to visualize the difference in measurements between the research nurse’s assessment and that of the care nurse and any trends in these differences in relation to the average NB care dependency score, the Bland-Altman figure was built.

All the analyses were carried out using the Statistical Package for the Social Sciences (SPSS) software, version 23.0, with the exception of Kp, which was calculated using the VassarStats electronic platform. A 5% significance level was adopted for all analyses.

### Ethical aspects

The research was approved by the Research Ethics Committee of the Pontifical Catholic University of Parana, under Opinion No. 5.127.442/2021. The people who agreed to take part in the study signed the Free and Informed Consent Form (ICF) for each group: nurses and parents/guardians of newborns.

## Results

Of the six nurses who took part, four had been working in neonatology for more than five years and reported having been trained to use the tool between one and three years ago; the other two nurses had been working in neonatology for less than five years and had not been trained to use the tool. 

The gestational age (GA) of the NBs admitted ranged from 29 to 39 weeks, with an average of NBs assessed at 34 weeks GA, with a standard deviation of three weeks more or less. The most frequent admission diagnoses were respiratory distress, with 49% (n = 17), and prematurity, with 23% (n = 8). 

As shown in Table 1, the areas of venous catheter control (95%), oxygenation (93%), weight and skin-mucosa integrity (92%) and vital signs control (90%) showed a high percentage of agreement (≥ 90%). When considering Kp, the items that showed strong or almost perfect agreement (Kp > 0.6) were: control of venous catheters (0.93), oxygenation (0.84), weight (0.82), thermoregulation (0.68) and body care (0.66).

**Table 1:** Distribution of the absolute and relative frequency of scores, the percentage of agreement between evaluators, Kp* and 95%CI† for the areas of care in the instrument (n‡ = 224). Curitiba, PR, Brazil, 2023

Area of care	1		2		3		Total		*K*p*	95%CI^†^
	n^‡^	%^§^	n^‡^	%^§^	n^‡^	%^§^	n^‡^	% agreement		
Thermoregulation	92	41	6	3	61	27	159	71	0,68	0,62 - 0,75
Weight	81	36	124	55	1	0	206	92	0,82	0,75 - 0,90
Spontaneous activity	89	40	23	10	6	3	118	53	0,21	0,12 - 0,30
Reaction to stimuli	94	42	16	7	2	1	112	50	0,15	0,08 - 0,23
Skin color	120	54	27	12	7	3	154	69	0,45	0,55 - 0,65
Tonicity	102	46	54	24	2	1	156	70	0,42	0,31 - 0,53
Elimination	188	84	5	2	0	0	193	86	0,19	0,03 - 0,35
Oxygenation	166	74	8	4	35	16	209	93	0,84	0,76 - 0,92
Skin-mucosa integrity	195	87	1	0	11	5	207	92	0,60	0,42 - 0,77
Body care	39	17	17	8	111	50	167	75	0,66	0,56 - 0,74
Control of vital signs	1	0	201	90	0	0	202	90	0,07	0,00 - 0,22
Control of probes and drains	62	28	26	12	9	4	97	43	0,40	0,35 - 0,47
Control of venous catheters	126	56	8	4	78	35	212	95	0,93	0,89 - 0,97
Drug therapy	56	25	39	17	25	11	120	54	0,44	0,36 - 0,52
Health education	112	50	20	9	8	4	140	63	0,29	0,19i - 0,39

*Kp = Weighted Kappa Coefficient; ^†^CI = Confidence Interval; ^‡^N = Absolute number; ^§^% = Percentage

Table 1 shows the Kp values for each of the instrument’s care areas. Strong agreement between the participants and low Kp values were found for the elimination and vital signs control areas. The areas of care weight, oxygenation and control of venous catheters showed an almost perfect degree of agreement. The area of care reaction to stimuli showed a weak degree of agreement.

**Figure 1:**
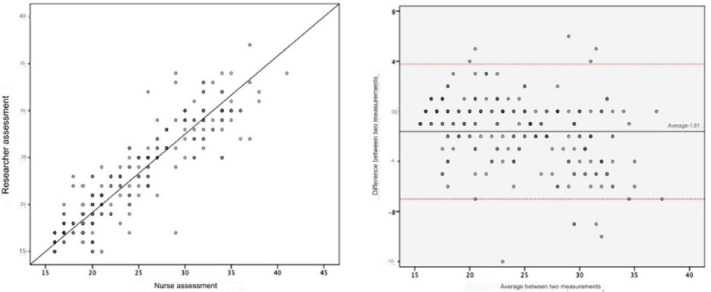
(a) Scatterplot of scores for care categorization carried out by the researcher and clinical nurses; (b) Bland- Altman figure for the difference and mean scores for care categorization carried out by the researcher and clinical nurses. Curitiba, PR, Brazil, 2023

The mean overall NB care dependency score of the research nurse was 23.57 (±5.23), while that of the nursing assistants was 25.19 (±6.16). The ICC between the overall NB care dependency score of the research nurse and the nursing assistants was 0.94, indicating strong reliability between the two measures (Figure 1a). As shown in the Bland-Altman figure, the mean difference between the measurements of the research nurse and the nursing assistants was -1.62 (±2.69), with a confidence interval (95%) of 5.19 points. There was no tendency for this difference to deviate according to the mean score (Figure 1b).

## Discussion

The quality of an instrument can be verified by analyzing the reliability criterion. Using this criterion, the instrument’s ability to generate consistent results for different users is observed^10^. Other researchers have carried out studies analyzing the reliability of instruments and the results are contributing to the quality and safety in the use of instruments, positively impacting care practice^11^ ^)-(^ ^14^.

The weight, oxygenation and catheter control areas of the instrument for classifying NBs according to the degree of dependence on nursing care analyzed in this research had almost perfect agreement, according to Kp.

Regarding weight, as it is a completely objective parameter, there was the expectation that agreement would be perfect between participants, which did not occur. This situation can be justified by the way the descriptions of the weight area scores are presented in the instrument. Score 3, for example, is indicated to score newborns who weigh more than 500 g or regardless of weight, which can cause difficulties in interpretation and decision-making, as there is a need to establish more precise limits. To minimize this limitation, the description could specify that the score covers weight > 500 g and < 1,000 g, making it more assertive and targeted. In the same sense, the description of score 2 could be changed to > 1,000 g and ≤ 2,500 g.

Measuring weight in neonatal units is an action frequently performed by the nursing team. The measurement guides important assessments, such as nutritional conditions and exposure to potential risks related to weight changes, which require organization of the nursing team’s care dynamics^6^. Therefore, it is essential to correctly record the weight in order to contribute to the score that will classify the newborn’s degree of dependence on care, directing care planning.

Considering the area of oxygenation care, in the same way, it is essential that the nurse adequately records clinical indicators of changes in the newborn’s breathing pattern. This can contribute, for example, to the rational use of oxygen therapies, consequently preventing possible complications^15^ ^)-(^ ^16^.

The oxygenation area of the instrument allows establishing a value that denotes the assessment of airway permeability and gas exchange hemostasis, with or without the aid of oxygen therapy^6^. Even with almost perfect agreement of evaluations between research participants, the descriptions of each score can generate doubts when choosing. Analysis of scores 2 and 3 in this area of the instrument (2 - NBs undergoing oxygen therapy in the incubator or via nasal catheter, halo or continuous nebulization; 3 - NBs undergoing halo, continuous nebulisation, nasal Continuous Positive Airway Pressure (CPAP) or ventilation pulmonary mechanics) leads to the possibility that a newborn may be in a halo and receive a score of 2, but may also receive a score of 3, given that the term halo is not specifically contextualized for any of the scores. The same occurs with newborns who use continuous nebulization. 

To adapt the situation of repetition of attributes in different scores, one possibility would be to specifically describe the support modalities to which each score refers. Thus, for example, for score 2, we suggest the description Nasal oxygen catheter, halo or continuous nebulization, while for score 3 it could be CPAP or mechanical ventilation. Furthermore, the logic of assigning a score of 2 or 3 to when the newborn is in a halo could be explained, which could contribute to minimizing doubts regarding score selection in this area, widely used and evaluated in the instrument. 

Likewise, intravenous therapy is frequently applied in the context of neonatology, demanding more time from the nursing team, both due to the number of medications to be prepared and infused and the attention and care throughout the process. In addition to its benefits, it can cause changes in well-being, pain, complications such as infection and extravasation in the skin^17^ ^)^ and anguish^18^. The nurse must be aware of the scientific evidence available to enhance positive effects and mitigate negative ones.

Even though the percentage of agreement among researchers stands out, the area of the venous catheter control instrument presents the possibility of different interpretations of alternatives related to scores. For example, if the nurse identifies a newborn with a catheter in an umbilical vessel, they may be in doubt as to which score to select on the instrument, since, for both score 2 and 3 in the area, there is a description of catheterization of umbilical vessels, without details of in which situation to select one score or another.

Under careful analysis, it is clear that the difference in the description for scores 2 and 3 consists of the expressions RN submitted and RN requiring umbilical vessel catheterization. This description can direct the interpretation in light of the dictionaryization of such terms. It can be interpreted that, for the NB submitted, a score of 2 is given, considering that it is the one that underwent catheterization of umbilical vessels due to secondary circumstances, such as difficulty in accessing a peripheral venous route.

For the newborn who needs it, a score of 3, considering that the umbilical vessels were catheterized for a previously analyzed purpose, such as the incompatibility of the osmolarity or pH of a medication with another route other than the one chosen or, even, a procedure such as exchange transfusion. As the descriptions can induce different interpretations, this can make it difficult to apply this area of the instrument and, consequently, the classification of the NB.

Although weaknesses are identified in the content of the instrument’s items that may hinder accurate interpretation, it allows nurses to evaluate complex and important items, as well as record them appropriately. The assessment and recording denote whether the NB is responding to the care received, allowing the analysis of results sensitive to nursing actions, which will determine the establishment of the workload and care planning. 

The newborn presents numerous responses that come from sensory, proprioceptive, biochemical, thermal and mechanical stimuli^6^, such as the perception of pain, which, although it can be perceived more attentively by the nursing team, has manifestations such as crying, which is not always related to pain itself but to other situations, such as emotional discomfort^19^. 

The weak agreement between participants regarding the reaction to stimuli can be explained by the difficulty in differentiating the descriptions of this area from those of the spontaneous activity area. The stimulus response area is described as the “capacity to respond to sensory, proprioceptive, biochemical, thermal and mechanical stimuli appropriate for successful extrauterine adaptation”^6^. 

In turn, the spontaneous activity area is described as the “ability to maintain the state of consciousness, the behavioral response to sensory, proprioceptive, biochemical, thermal and mechanical stimuli and physiological parameters suitable for a successful extrauterine adaptation”^6^. It is noted that the terms ability to respond (related to the identification of the NB’s responses) and ability to maintain (related to the identification of the NB’s maintenance parameters) can be easily confused, even though they have different meanings.

Although the areas of elimination and control of vital signs showed a considerable percentage of agreement (> 85%), when determining the Kp value, low reliability was identified. This inconsistency is due to the lack of answers for one of the categories, either by the nurse researcher or by the clinical nurses. Kappa is a method recommended for square matrices, that is, the two variables to be analyzed must have the same number of categories. However, in this study, the small variability of responses in these two items meant that some response options were not recorded; therefore, the square matrix assumption of the Kappa index was violated, making its interpretation unfeasible. Therefore, for interpretation purposes, it seems prudent, in these cases, to base conclusions on the percentage of agreement, until studies with more heterogeneous assessments are carried out.

This situation can be explained by the fact that participants did not diversify their answers, focusing them on just two options. In this case, the options that make up the eliminations area of the instrument allow you to determine whether the newborn has autonomy and/or dysfunction of the digestive and/or genitourinary system. Score 3 is assigned to the NB who presents changes in elimination patterns, with ostomies, control of eliminations by diaper weight and/or presence of a bladder catheter and/or collection bag, and no NB evaluated in this research required interventions such as bladder catheterization or elimination ostomy. 

This also happened with the results related to the vital signs area, given that assessments for the area require significant modifications to be identified in the instrument. In the context of the NICU, vital signs are assessed every four hours, along with other care provided, avoiding unnecessary handling of the newborn.

Although variations were identified in the assessments of the instrument’s care areas, this did not reduce the reliability of the assessment for defining the categorization of care (minimum, intermediate and intensive) through its use. This was identified by comparing the sum of care areas, which revealed a strong correlation between research participants (ICC of 0.94). However, it is suggested that the application of the instrument be accompanied by a manual, which can minimize subjectivity in the interpretation of the instrument’s content and application^20^. Such manual must be prepared with detailed explanations of terms and their contexts in the instrument, in addition to certification of the training carried out.

As limitations of the research, it can be related to the fact that other psychometric properties of the instrument were not measured and that it was carried out during the period of the coronavirus disease-2019 (COVID-19) pandemic, which weakened the opportunities for the presence and participation of parents and /or responsible for activities with NBs in the unit. This limitation interfered with the evaluation of the health education area, given the need for family participation in a significant way in the neonatal environment so that nurses can evaluate this area of care, considering experiences and understanding about care actions and shared information. 

The nurse has the primary role of acting as a facilitator with the family, equipping them to provide care associated with the needs of the newborn and promoting the formation of an emotional bond between parents and newborn^21^.

The application of an instrument that assesses the degree of dependence on nursing care allows the identification of client needs that will be relevant for planning care, as well as data on the workload, which will favor the adequate dimensioning of the nursing team. The use of an instrument can help nurses, even with unequal degrees of expertise, plan appropriate nursing care for different clients and/or contexts. 

## Conclusion

Among the 15 areas of the instrument for classifying NBs according to the degree of dependence on nursing care, there was almost perfect agreement for the areas of weight care, oxygenation and control of venous catheters. Agreement was weak for the stimulus reaction area. The instrument is reliable for classifying NBs according to the degree of dependence on nursing care in the NICU, with an ICC of 0.94.

Although there were variations in the assessments of the areas of care proposed by the instrument, this did not reduce the reliability of the instrument in categorizing the type of neonatal care, classified as minimum, intermediate and intensive. It is suggested that the use of the instrument be accompanied by a manual containing the definition of terms that require interpretation and/or contextualization for care scenarios. 
