# Fe de Erratas: Instrumento para clasificación de dependencia de cuidados neonatales intensivos: análisis de concordancia y de confianza*

**DOI:** 10.1590/1518-8345.0000.4739

**Published:** 2025-09-05

En el artículo “Instrumento para clasificación de dependencia de cuidados neonatales intensivos: análisis de concordancia y de confianza”, con número DOI: 10.1590/1518-8345.6922.4271, publicado en la Rev. Latino-Am. Enfermagem, 2024;32:e4271, en la página 1:

Donde se leía:

“DOI: 10.1590/1518-8345.6761.4171”

Leer:

“DOI: 10.1590/1518-8345.6922.4271”

