# Supplementary material for: Instrument for classifying dependency in neonatal intensive care: analysis of agreement and reliability
Source: Rev Lat Am Enfermagem. 2024 Aug 30;32:e4272. doi: 10.1590/1518-8345.6922.4272 (PMC11368069; doi:10.1590/1518-8345.6922.4272)
Supplement: Translation

# Translation: Instrumento para classificação de dependência de cuidados neonatais intensivos: análise de concordância e de confiança*

**DOI:** 10.1590/1518-8345.6922.4273

**Authors:** Paula Mara Bordim Lopes, Fernanda Broering Gomes Torres, Thomaz Jefferson Massaneiro, Adriano Akira Hino, Elenice Valentim Carmona, Marcia Regina Cubas

**Affiliations:** 1 Pontifícia Universidade Católica do Paraná, Curitiba, Paraná, PR, Brasil.; 2 Pontifícia Universidade Católica do Paraná, Escola de Medicina, Curitiba, Paraná, PR, Brasil.; 3 Universidade Estadual de Campinas, Faculdade de Enfermagem, Campinas, São Paulo, SP, Brasil.

**Keywords:** Avaliação em Saúde, Enfermagem Neonatal, Estudos de Validação, Unidades de Terapia Intensiva Neonatal, Cuidados de Enfermagem, Planejamento de Assistência ao Paciente

## Abstract

**Objetivo:**

analisar a confiabilidade dos itens que compõem o instrumento para classificação de recém-nascido de acordo com o grau de dependência dos cuidados de enfermagem em uma unidade de terapia intensiva neonatal.

**Método:**

estudo metodológico que analisou a concordância e a confiança do instrumento em uma unidade de terapia intensiva neonatal. Seis enfermeiros assistenciais e uma enfermeira pesquisadora avaliaram 35 recém-nascidos e preencheram o instrumento, composto por 15 áreas de cuidado. O coeficiente Kappa ponderado e o Coeficiente de Correlação Intraclasse foram utilizados para análise.

**Resultados:**

as áreas peso (92%), oxigenação (93%) e controle de cateteres (95%) obtiveram concordância quase perfeita, e a área reação a estímulos (50%) obteve concordância fraca. As áreas eliminações e sinais vitais apresentaram baixa confiabilidade, devido à pouca variabilidade de respostas. O Coeficiente de Correlação Intraclasse foi de 0,94.

**Conclusão:**

há variações nas avaliações de algumas áreas de cuidado devido à descrição imprecisa de itens aos quais são atribuídos escores, entretanto, o instrumento é confiável para categorização do tipo de cuidado (mínimo, intermediário e intensivo). Sua utilização pode contribuir para o dimensionamento de qualidade e para a segurança da assistência ao recém-nascido.

## Destaques

**(1)** O instrumento é confiável para categorização do tipo de cuidado ao recém-nascido.

**(2)** A utilização de um manual pode minimizar a subjetividade do instrumento.

**(3)** Há concordância quase perfeita em áreas menos subjetivas do instrumento.

## Introdução

Anualmente, nascem mais de 15 milhões de prematuros no mundo^1^, sendo a prematuridade considerada a principal causa de admissões em Unidade de Terapia Intensiva Neonatal (UTIN). Além da prematuridade e suas complicações, outras situações clínicas do período neonatal requerem cuidados intensivos. 

Assim, a UTIN é destinada ao Recém-Nascido (RN) que requer cuidados complexos em diferentes graus e tem como finalidade oferecer assistência à saúde, com recursos tecnológicos, profissionais de qualidade e segurança^2^. O enfermeiro neonatologista precisa identificar necessidades específicas de cada RN para o planejamento e organização da assistência de enfermagem segura e de qualidade. Nesse sentido, algumas ferramentas podem apoiar os enfermeiros neonatologistas na identificação de resultados de ações na prática clínica^3^, na avaliação da complexidade da carga de trabalho cognitiva de enfermagem^4^ *,* na mensuração da intensidade dos tratamentos e para o dimensionamento da equipe de enfermagem^5^.

Com o objetivo de classificar o RN de acordo com o grau de dependência de cuidados de enfermagem, em 2000, foi elaborado um instrumento voltado para o perfil de RN em UTIN^6^. Inicialmente, o instrumento contemplava 16 áreas de cuidado: termorregulação, peso, atividade espontânea, reação a estímulos, cor da pele, tonicidade muscular, nutrição e hidratação, oxigenação, integridade cutânea mucosa, temperatura, frequência respiratória, frequência cardíaca, pressão arterial, saturação de oxigênio, controle de drenos, sondas e cateteres, e terapêutica medicamentosa.

Em 2005, o instrumento foi reestruturado para o contexto brasileiro, de modo a adequá-lo às necessidades individualizadas de cuidado, sendo retirada a área nutrição e hidratação. Assim, a versão destinada ao uso no Brasil é composta por 15 áreas de cuidado^6^, sendo essa a versão utilizada neste estudo.

Apesar de o instrumento de avaliação e classificação de RN ter passado por um processo de validação de conteúdo, não foi identificado estudo que tenha investigado sua confiabilidade na prática clínica, o que é incentivado pelas autoras do instrumento^6^. A análise de confiabilidade de instrumentos implementados na prática assistencial é fundamental para a segurança da aplicação do instrumento, propor ajustes e dimensionar os cuidados de enfermagem ao recém-nascido^3^ ^)-(^ ^5^.

Desse modo, com o intuito de contribuir com o processo de validação do instrumento, o objetivo desta pesquisa foi analisar a confiabilidade dos itens que compõem o instrumento para classificação de RN de acordo com o grau de dependência dos cuidados de enfermagem em uma UTIN.

## Método

### Tipo do estudo

Trata-se de um estudo metodológico que analisou a concordância e a confiança de instrumento para classificação de recém-nascido de acordo com o grau de dependência dos cuidados de enfermagem em uma unidade de terapia intensiva neonatal.

### Local da coleta de dados

O estudo foi realizado na UTIN de um hospital privado na cidade de Curitiba, Paraná, Brasil. Nesse hospital, o instrumento para classificação de RN, de acordo com o grau de dependência de cuidados de enfermagem^6^, é utilizado na rotina assistencial, tendo sido incorporado ao sistema de registro eletrônico do paciente em 2017.

### Período

A coleta de dados deste estudo ocorreu no período de dezembro de 2021 a fevereiro de 2022.

### População

Participaram do estudo seis enfermeiros da UTIN, e 35 RNs admitidos no setor no período da coleta. Na UTIN do hospital, os principais motivos para internação dos RNs foram a prematuridade e/ou desconforto respiratório

### Critérios de seleção

Todos os enfermeiros do setor foram selecionados para participar da pesquisa, tendo como critério de inclusão ser especialista em neonatologia, enquanto o de exclusão foi estar em férias ou afastado durante o período de coleta de dados. Todos os RNs admitidos no setor durante o período de coleta e cujos pais aceitaram participar da pesquisa foram incluídos no estudo, sem critério de exclusão. 

### Definição da amostra

A amostra foi probabilística, sistemática, em que se considerou uma proporção mínima de 210 avaliações e máxima de 420 avaliações do conjunto de RNs participantes. Foram realizadas 224 avaliações. A média de avaliação por RN foi de oito dias consecutivos.

### Variáveis do estudo

As variáveis do estudo compreendem as áreas do instrumento para classificação de RN^6^, são elas:

- Termorregulação - capacidade de manutenção da temperatura corporal estável, com gasto calórico e consumo de oxigênio mínimos para uma adaptação extrauterina bem-sucedida;
- Peso - necessidade de controle de peso para comparar o peso diário com o peso ao nascer e com o peso do dia anterior, auxiliando na avaliação das condições nutricionais e riscos potenciais decorrentes do peso de nascimento;
- Atividade espontânea - habilidade em manter o estado de consciência, a resposta comportamental aos estímulos sensoriais, proprioceptivos, bioquímicos, térmicos e mecânicos e parâmetros fisiológicos adequados para uma adaptação extrauterina bem-sucedida;
- Reação a estímulos - capacidade de responder a estímulos sensoriais, proprioceptivos, bioquímicos, térmicos e mecânicos adequados para uma adaptação extrauterina bem-sucedida;
- Cor da pele - capacidade de manter pele e mucosas coradas adequadas para uma adaptação extrauterina bem-sucedida;
- Tonicidade - capacidade de manter-se com tônus muscular vigoroso adequado a uma adaptação extrauterina bem-sucedida;
- Eliminações - habilidade em manter eliminações urinária e intestinal espontâneas com auxílio de terceiros ou por drenos e estoma;
- Oxigenação - aptidão em manter a permeabilidade das vias aéreas e o equilíbrio nas trocas gasosas por si mesmo ou com auxílio da equipe de enfermagem e/ou de equipamentos;
- Integridade cutâneo-mucosa - capacidade de manter pele e mucosas sem danificação ou destruição;
- Cuidado corporal - capacidade de manter a higiene pessoal, vestuário;
- Controle de sinais vitais (SSVV) - necessidade de observação e controles dos parâmetros vitais - temperatura (T), frequência respiratória (FR), frequência cardíaca (FC), pressão arterial (PA), saturação de O2_;_
- Controle de sondas e drenos - necessidade de observação e controle dos equipamentos contendo fluidos de infusão e/ou drenagem;
- Controle de cateteres venosos - necessidade de observação e controle dos cateteres de infusão e/ou coletas, monitorização hemodinâmica e nutrição parenteral hipertônica;
- Terapêutica medicamentosa - utilização dos diversos medicamentos terapêuticos (drogas, soluções, sangue e hemoderivados);
- Educação à saúde - habilidade, confiança e segurança da mãe/família para dispensar cuidados adequados para a manutenção dos hábitos de saúde pessoais e/ou ambientais dos RNs.

### Instrumentos utilizados para a coleta das informações

O instrumento utilizado para avaliação e classificação de RN^6^ é composto por 15 áreas de cuidado, contendo uma questão para cada área e sendo atribuído peso de um a três para cada item. O peso um corresponde ao menor grau de dependência de cuidados de enfermagem e o peso três, ao maior^6^. Para fins de análise do escore geral de dependência de cuidados de enfermagem, todas as respostas são somadas, resultando em uma pontuação mínima de 15 e máxima de 45 pontos. A somatória dos pontos corresponde a três categorias de cuidado: mínimo (15 a 25 pontos), intermediário (26 a 36 pontos) e intensivo (37 a 45 pontos). Para padronização dos escores, as autoras do instrumento definiram o significado de cada categoria e atribuíram a distribuição da pontuação, levando em conta a instabilidade clínica do RN e as respostas oferecidas pelos juízes, após aplicação de técnica Delphi, com índice de concordância acima de 70%^6^.

Houve comunicação entre a autora principal do instrumento de classificação^6^ e a primeira autora do presente manuscrito para apresentação do objetivo da pesquisa e esclarecimentos. Em virtude da publicação do instrumento na íntegra, não houve autorização formal, apenas ciência do uso.

### Coleta de dados

Os 35 RNs foram avaliados diariamente, duplamente e no mesmo momento pela enfermeira pesquisadora e por um enfermeiro assistencial, com o preenchimento do instrumento concomitante à avaliação. A pesquisadora não interferiu na avaliação do enfermeiro. Essas estratégias foram utilizadas para minimizar a coleta de dados entre os avaliadores.

### Tratamento e análise dos dados

Para fins de descrição dos 15 itens que contemplam as áreas de cuidado, calculou-se a distribuição de frequências absoluta e relativa de cada área de cuidado em que houve concordância entre a enfermeira pesquisadora e o enfermeiro assistencial. O mesmo foi realizado para o total de casos concordantes (n e % de concordância). 

Para a avaliação da confiabilidade de cada item do instrumento, foi utilizado o coeficiente Kappa ponderado (*K*p), considerando que as opções de respostas não eram dicotômicas^7^_._ O *K*p varia de 0 a 1, sendo que, quanto mais próximo de 1, maior é o indicativo de concordância entre os avaliadores. Para a interpretação do valor de *K*p, os intervalos correspondentes foram classificados da seguinte forma: menor que 0,00 - concordância insignificante; de 0,00 a 0,20 - concordância fraca; de 0,21 a 0,40 - concordância razoável; de 0,41 a 0,60 - concordância moderada; de 0,61 a 0,80 - concordância forte; e de 0,81 a 1,00 - concordância quase perfeita^8^. 

Para avaliação da confiabilidade do escore geral de dependência de cuidados de RNs gerado pela soma de todas as respostas, utilizou-se o Coeficiente de Correlação Intraclasse (CCI). Para fins de interpretação dele, empregou-se a seguinte classificação: menor que 0,5 - baixa confiabilidade; de 0,5 a 0,75 - confiabilidade moderada; de 0,75 a 0,90 - boa confiabilidade; e maior que 0,90 - excelente confiabilidade^9^. 

Por fim, para a visualização da diferença nas medidas entre a avaliação da enfermeira pesquisadora e do enfermeiro assistencial e eventuais tendências dessas diferenças em relação à média do escore de dependência de cuidados de RNs, foi construído o gráfico de Bland-Altman.

Todas as análises foram realizadas com o auxílio do *software Statistical Package for the Social Sciences* (SPSS), versão 23.0, com exceção do *K*p, que foi calculado com o auxílio da plataforma eletrônica *VassarStats*. Em todas as análises, foi adotado o nível de significância de 5%.

### Aspectos éticos

A pesquisa foi aprovada pelo Comitê de Ética em Pesquisa da Pontifícia Universidade Católica do Paraná, sob o Parecer nº 5.127.442/2021. As pessoas que concordaram em participar do estudo assinaram o Termo de Consentimento Livre e Esclarecido (TCLE) direcionado a cada grupo: enfermeiros e pais/responsáveis pelo RN.

## Resultados

Dos seis enfermeiros participantes, quatro atuavam há mais de cinco anos na área de neonatologia e relataram ter realizado capacitação para o uso do instrumento há cerca de um a três anos; os outros dois enfermeiros atuavam há menos de cinco anos na área de neonatologia e não tiveram capacitação para a aplicação do instrumento. 

A Idade Gestacional (IG) dos RNs admitidos variou de 29 a 39 semanas, tendo uma média de RNs avaliados com 34 semanas de IG, com desvio-padrão de três semanas para mais ou para menos. Os diagnósticos de admissão mais frequentes foram desconforto respiratório, com 49% (n = 17), e prematuridade, com 23% (n = 8). 

Conforme apresentado na Tabela 1, as áreas controle de cateteres venosos (95%), oxigenação (93%), peso e integridade cutâneo-mucosa (92%) e controle de sinais vitais (90%) apresentaram um percentual de concordância alto (≥ 90%). Ao considerar o *K*p, os itens que apresentaram concordância forte ou quase perfeita (*K*p > 0,6) foram: controle de cateteres venosos (0,93), oxigenação (0,84), peso (0,82), termorregulação (0,68) e cuidado corporal (0,66).

**Tabela 1:** Distribuição da frequência absoluta e relativa dos escores, da porcentagem de concordância entre avaliadores, *K*p* e IC^†^ 95% para as áreas de cuidado do instrumento (n^‡^ = 224). Curitiba, PR, Brasil, 2023

Área de cuidado	1		2		3		Total		*K*p*	IC^†^ 95%
	n^‡^	%^§^	n^‡^	%^§^	n^‡^	%^§^	n^‡^	% de concordância		
Termorregulação	92	41	6	3	61	27	159	71	0,68	0,62 - 0,75
Peso	81	36	124	55	1	0	206	92	0,82	0,75 - 0,90
Atividade espontânea	89	40	23	10	6	3	118	53	0,21	0,12 - 0,30
Reação a estímulos	94	42	16	7	2	1	112	50	0,15	0,08 - 0,23
Cor da pele	120	54	27	12	7	3	154	69	0,45	0,55 - 0,65
Tonicidade	102	46	54	24	2	1	156	70	0,42	0,31 - 0,53
Eliminações	188	84	5	2	0	0	193	86	0,19	0,03 - 0,35
Oxigenação	166	74	8	4	35	16	209	93	0,84	0,76 - 0,92
Integridade cutâneo-mucosa	195	87	1	0	11	5	207	92	0,60	0,42 - 0,77
Cuidado corporal	39	17	17	8	111	50	167	75	0,66	0,56 - 0,74
Controle de sinais vitais	1	0	201	90	0	0	202	90	0,07	0,00 - 0,22
Controle de sondas e drenos	62	28	26	12	9	4	97	43	0,40	0,35 - 0,47
Controle de cateteres venosos	126	56	8	4	78	35	212	95	0,93	0,89 - 0,97
Terapêutica medicamentosa	56	25	39	17	25	11	120	54	0,44	0,36 - 0,52
Educação em saúde	112	50	20	9	8	4	140	63	0,29	0,19i - 0,39

*Kp = Coeficiente Kappa ponderado; ^†^IC = Intervalo de Confiança; ^‡^N = Número absoluto; ^§^% = Porcentagem

Na Tabela 1, apresentam-se os valores do *K*p para cada uma das áreas de cuidado do instrumento. Identificou-se forte concordância entre os participantes e valor baixo de *K*p para as áreas eliminações e controle de sinais vitais. As áreas de cuidado peso, oxigenação e controle de cateteres venosos mostraram grau de concordância quase perfeita. A área de cuidado reação a estímulos revelou grau de concordância fraca.

A média do escore geral de dependência de cuidados de RNs da enfermeira pesquisadora foi de 23,57(±5,23), enquanto a dos enfermeiros assistenciais foi de 25,19(±6,16). O CCI entre o escore geral de dependência de cuidados de RNs da enfermeira pesquisadora e dos enfermeiros assistenciais foi de 0,94, indicando forte confiabilidade entre as duas medidas (Figura 1a). Conforme apresentado no gráfico de Bland-Altman, a diferença média entre as medidas da enfermeira pesquisadora e dos enfermeiros assistenciais foi de -1,62(±2,69), com intervalo de confiança (95%) contido entre 5,19 pontos. Não foi observada tendência de desvio dessa diferença, de acordo com a média do escore (Figura 1b).

**Figura 1:** (a) Gráfico de dispersão das pontuações para categorização de cuidado realizadas pela pesquisadora e pelos enfermeiros assistenciais; (b) Gráfico de Bland-Altman para a diferença e a média das pontuações para categorização de cuidado realizadas pela pesquisadora e pelos enfermeiros assistenciais. Curitiba, PR, Brasil, 2023 As áreas relacionadas ao peso, oxigenação e controle de cateteres do instrumento para classificação de RNs, de acordo com o grau de dependência de cuidados de enfermagem analisado, nesta pesquisa, tiveram concordância quase perfeita, segundo o *K*p

## Discussão

A qualidade de um instrumento pode ser verificada por meio da análise do critério de confiabilidade. Por esse critério, observa-se a capacidade do instrumento em gerar resultados consistentes por distintos usuários^10^. Outros pesquisadores realizaram estudos de análise de confiabilidade de instrumentos e os resultados estão contribuindo com a qualidade e segurança na utilização de instrumentos, implicando positivamente na prática assistencial^11^ ^)-(^ ^14^.

Em relação ao peso, por se tratar de um parâmetro totalmente objetivo, havia a expectativa de que a concordância fosse perfeita entre os participantes, o que não ocorreu. Tal situação pode ser justificada pela forma como as descrições das pontuações da área peso estão apresentadas no instrumento. O escore 3, por exemplo, é indicado para pontuar o RN que tenha peso superior a 500 g ou independentemente do peso, o que pode causar dificuldade de interpretação e tomada de decisão, visto que há necessidade de estabelecimento de limites mais precisos. Para minimizar essa limitação, a descrição poderia especificar que o escore abrange peso > 500 g e < 1.000 g, deixando-a mais assertiva e direcionada. No mesmo sentido, a descrição do escore 2 poderia ser alterada para > 1.000 g e ≤ 2.500 g.

A mensuração do peso em unidades neonatais é uma ação frequentemente executada pela equipe de enfermagem. A mensuração pauta avaliações importantes, como as condições nutricionais e exposição a potenciais riscos relacionados às alterações do peso, as quais demandam organização da dinâmica de cuidados da equipe de enfermagem^6^. Sendo assim, é imprescindível o correto registro do peso a fim de contribuir com o escore que classificará o grau de dependência de cuidados do RN, direcionando o planejamento da assistência.

Ao considerar a área de cuidado oxigenação, da mesma forma, é imprescindível que o enfermeiro registre adequadamente indicadores clínicos de alteração do padrão respiratório do RN. Isso pode contribuir, por exemplo, para o uso racional de oxigenoterapias, consequentemente prevenindo possíveis complicações^15^ ^)-(^ ^16^.

A área *oxigenação* do instrumento permite estabelecer um valor que denota a avaliação da permeabilidade das vias aéreas e hemostasia das trocas gasosas, com ou sem auxílio de oxigenoterapia^6^. Mesmo com concordância quase perfeita de avaliações entre os participantes da pesquisa, as descrições de cada escore podem gerar dúvidas no momento da escolha. A análise dos escores 2 e 3 dessa área do instrumento (2 - RNs submetidos à oxigenoterapia na incubadora ou por cateter nasal, halo ou nebulização contínua; 3 - RNs submetidos a halo, nebulização contínua, *Continuous Positive Airway Pressure* (CPAP) nasal ou ventilação pulmonar mecânica leva à possibilidade de que um RN pode estar em halo e receber pontuação 2, mas também pode receber a pontuação 3, dado que o termo halo não é contextualizado especificamente para nenhum dos escores. O mesmo ocorre com o RN que faz uso de nebulização contínua. 

Para adequar a situação de repetição de atributos em escores diferentes, uma possibilidade seria a descrição específica das modalidades de suporte a que se refere cada pontuação. Assim, sugere-se, por exemplo, para o escore 2, a descrição Cateter nasal de oxigênio, halo ou nebulização contínua, enquanto, para o 3, poderia ser CPAP ou ventilação mecânica. Ainda, pode-se explicitar a lógica de designar a situação de escore 2 ou 3 para quando o RN estiver em halo, o que poderia contribuir para a minimização de dúvida de seleção de escore dessa área amplamente utilizada e avaliada no instrumento. 

Da mesma forma, a terapia endovenosa é frequentemente aplicada ao contexto da neonatologia, demandando maior tempo da equipe de enfermagem, tanto em função do número de medicamentos a ser preparados e infundidos quanto da atenção e cuidado ao longo do processo. Além de seus benefícios, ela pode implicar alterações do bem-estar, dor, complicações como infecção e extravasamento na pele^17^ ^)^ e angústia^18^. O enfermeiro deve estar atento às evidências científicas disponíveis para potencializar efeitos positivos e atenuar os negativos. 

Mesmo tendo destaque na porcentagem de concordância entre os pesquisadores, a área do instrumento controle de cateteres venosos apresenta possibilidade de diferentes interpretações das alternativas relacionadas aos escores. Por exemplo, se o enfermeiro identifica um RN com cateter em vaso umbilical, ele pode ficar em dúvida de qual escore selecionar no instrumento, pois, tanto para o escore 2 quanto para o 3 da área, há a descrição de cateterização de vasos umbilicais, sem detalhamento de em qual situação selecionar uma pontuação ou outra. 

Sob análise atenta, percebe-se que a diferença na descrição para as pontuações 2 e 3 consiste nas expressões RN submetido e RN que necessita de cateterização de vasos umbilicais. Essa descrição pode direcionar a interpretação à luz da dicionarização de tais termos. Pode-se interpretar que, para o RN submetido, pontua-se o escore 2, considerando que se trata daquele que foi subordinado à cateterização de vasos umbilicais devido à circunstância secundária, como a dificuldade de acessar uma via venosa periférica. 

Já para o RN que necessita, pontua-se o escore 3, considerando que teve cateterizados os vasos umbilicais para determinado fim previamente analisado, como a incompatibilidade da osmolaridade ou pH de um medicamento com outra via que não a escolhida ou, ainda, procedimento como exsanguineotransfusão. Como as descrições podem induzir diferentes interpretações, isso pode trazer dificuldade para a aplicação dessa área do instrumento e, consequentemente, para a classificação do RN. 

Embora sejam identificadas fragilidades do conteúdo de itens do instrumento que podem dificultar a interpretação acurada, ele permite que o enfermeiro avalie complexas e importantes situações, bem como as registre de forma adequada. A avaliação e o registro denotam se o RN está respondendo ao cuidado recebido, permitindo analisar os resultados sensíveis às ações da enfermagem, o que determinará o estabelecimento da carga de trabalho e do planejamento da assistência. 

O RN apresenta inúmeras respostas que são oriundas de estímulos sensoriais, proprioceptivos, bioquímicos, térmicos e mecânicos^6^, a exemplo da percepção da dor que, embora possa ser percebida de forma mais atenta pela equipe de enfermagem, tem manifestações como o choro, estando este nem sempre relacionado à dor propriamente e sim a outras situações, como desconforto emocional^19^. 

A fraca concordância entre os participantes em relação à reação a estímulos pode ser explicada pela dificuldade de diferenciar as descrições desta área daquelas da área de atividade espontânea. Descreve-se a área reação a estímulos como a “capacidade de responder a estímulos sensoriais, proprioceptivos, bioquímicos, térmicos e mecânicos adequados para uma adaptação extrauterina bem-sucedida”^6^. 

Por sua vez, a área atividade espontânea é descrita como a “habilidade de manter o estado de consciência, a resposta comportamental aos estímulos sensoriais, proprioceptivos, bioquímicos, térmicos e mecânicos e parâmetros fisiológicos adequados para uma adaptação extrauterina bem-sucedida”^6^. Nota-se que os termos capacidade de responder (relacionado com a identificação de respostas do RN) e habilidade de manter (relacionado com a identificação de parâmetros de manutenção do RN) podem ser facilmente confundidos, ainda que tenham significados diferentes.

Embora as áreas eliminações e controle de sinais vitais tenham apresentado considerável percentual de concordância (> 85%), pela determinação do valor de *K*p, foi identificada baixa confiabilidade. A inconsistência se deve à ausência de respostas para uma das categorias, seja pela pesquisadora enfermeira, seja pelos enfermeiros assistenciais. O Kappa é um método indicado para matrizes quadradas, ou seja, as duas variáveis a serem analisadas devem possuir o mesmo número de categorias. No entanto, neste estudo, a pequena variabilidade das respostas nesses dois itens fez com que algumas opções de resposta não obtivessem registro; logo, o pressuposto de matriz quadrada do índice Kappa foi violado, inviabilizando sua interpretação. Dessa forma, para fins de interpretação, parece ser prudente, nesses casos, basear as conclusões no percentual de concordância, até que estudos com avaliações mais heterogêneas sejam realizados.

Tal situação pode ser explicada pelo fato de os participantes não diversificarem suas respostas, concentrando-as em apenas duas opções. No caso, as opções que compõem a área eliminações do instrumento permitem pontuar se o RN apresenta autonomia e/ou disfunção do sistema digestório e/ou geniturinário. O escore 3 é designado para o RN que apresenta alterações nos padrões das eliminações, com ostomias, controle das eliminações por peso de fralda e/ou presença de cateter vesical e/ou saco coletor, sendo que nenhum RN avaliado nesta pesquisa precisou de intervenções como cateterismo vesical ou estomia de eliminação. 

Isso também aconteceu com os resultados relacionados à área sinais vitais, dado que as avaliações para a área requerem modificações expressivas para serem identificadas no instrumento. No contexto da UTIN, os sinais vitais são avaliados a cada quatro horas, junto de outros cuidados prestados, evitando manuseio desnecessário do RN. 

Embora tenham sido identificadas variações nas avaliações das áreas de cuidado do instrumento, isso não reduziu a confiabilidade da avaliação para definir a categorização do cuidado (mínimo, intermediário e intensivo) por meio de sua utilização. Isso foi identificado pela comparação da somatória das áreas de cuidado, que revelou uma forte correlação entre os participantes da pesquisa (CCI de 0,94). Entretanto, sugere-se que a aplicação do instrumento seja acompanhada por um manual, que pode minimizar a subjetividade na interpretação do conteúdo do instrumento e aplicação^20^. Tal manual deve ser elaborado com explicações detalhadas de termos e seus contextos no instrumento, além da certificação da capacitação realizada.

Como limitações da pesquisa, pode-se indicar o fato que não foram mensuradas outras propriedades psicométricas do instrumento e que foi realizada no período da pandemia de *coronavirus disease-2019* (COVID-19), o que fragilizou as oportunidades de presença e participação de pais e/ou responsáveis em atividades junto aos RNs na unidade. Essa limitação interferiu na avaliação da área educação à saúde, diante da necessidade da participação da família de maneira expressiva no ambiente neonatal para que o enfermeiro possa avaliar essa área de cuidado, considerando vivências e compreensão sobre ações de cuidado e informações compartilhadas. 

O enfermeiro tem o papel primordial de atuar como facilitador junto à família, instrumentalizando-a para os cuidados associados às necessidades do RN e favorecendo a formação do vínculo afetivo entre pais e RN^21^.

A aplicação de um instrumento que avalie o grau de dependência dos cuidados de enfermagem permite que sejam identificadas necessidades da clientela que serão relevantes para o planejamento da assistência, bem dados sobre a carga de trabalho, o que favorecerá o adequado dimensionamento da equipe de enfermagem. O uso de um instrumento pode contribuir para que enfermeiros, mesmo com desiguais graus de *expertise*, planejem adequada assistência de enfermagem para diferentes clientes e/ou contextos. 

## Conclusão

Dentre as 15 áreas do instrumento para classificação de RN de acordo com o grau de dependência dos cuidados de enfermagem, verificou-se concordância quase perfeita para as áreas de cuidado peso, oxigenação e controle de cateteres venosos. A concordância foi fraca para a área reação a estímulos. O instrumento é confiável para classificar RN de acordo com o grau de dependência dos cuidados de enfermagem em UTIN, com CCI de 0,94. 

Embora tenham ocorrido variações nas avaliações das áreas de cuidado propostas pelo instrumento, isso não reduziu a confiabilidade do instrumento na categorização do tipo de cuidado neonatal, classificado em mínimo, intermediário e intensivo. Sugere-se que a utilização do instrumento seja acompanhada por um manual contendo a definição de termos que requerem interpretação e/ou contextualização para os cenários assistenciais. 

# Translation: Instrumento para clasificación de dependencia de cuidados neonatales intensivos: análisis de concordancia y de confianza*

**DOI:** 10.1590/1518-8345.6922.4271

**Authors:** Paula Mara Bordim Lopes, Fernanda Broering Gomes Torres, Thomaz Jefferson Massaneiro, Adriano Akira Hino, Elenice Valentim Carmona, Marcia Regina Cubas

**Affiliations:** 1 Pontifícia Universidade Católica do Paraná, Curitiba, Paraná, PR, Brasil.; 2 Pontifícia Universidade Católica do Paraná, Escola de Medicina, Curitiba, Paraná, PR, Brasil.; 3 Universidade Estadual de Campinas, Faculdade de Enfermagem, Campinas, São Paulo, SP, Brasil.

**Keywords:** Valoración de Salud, Enfermería Neonatal, Estudios de Validación, Unidades de Cuidados Intensivos Neonatales, Cuidado de Enfermera, Planificación de Atención al Paciente

## Abstract

**Objetivo:**

analizar la confiabilidad de los ítems que componen el instrumento para clasificación de recién nacido de acuerdo con el grado de dependencia de los cuidados de enfermería en una unidad de terapia de cuidados intensivos neonatal.

**Método:**

estudio metodológico que analizó la concordancia y la confianza del instrumento en una unidad de terapia de cuidados intensivos neonatal. Seis enfermeros asistenciales y una enfermera investigadora evaluaron 35 recién nacidos y completaron el instrumento, compuesto por 15 áreas de cuidado. El coeficiente Kappa ponderado y el Coeficiente de Correlación Intraclase fueron utilizados para el análisis.

**Resultados:**

las áreas peso (92%), oxigenación (93%) y control de catéteres (95%) obtuvieron una concordancia casi perfecta y el área reacción a estímulos (50%) obtuvo una concordancia débil. Las áreas eliminaciones y signos vitales presentaron baja confiabilidad, debido a la poca variabilidad de respuestas. El Coeficiente de Correlación Intraclase fue de 0,94.

**Conclusión:**

hay variaciones en las evaluaciones de algunas áreas de cuidado debido a la descripción imprecisa de ítems a los cuales se les atribuyen puntuaciones, sin embargo, el instrumento es confiable para la categorización del tipo de cuidado (mínimo, intermedio e intensivo). Su utilización puede contribuir a la planificación de calidad y a la seguridad de la atención al recién nacido.

## Destacados

**(1)** El instrumento es confiable para la categorización del tipo de cuidado al recién nacido.

**(2)** La utilización de un manual puede minimizar la subjetividad del instrumento.

**(3)** Hay una concordancia casi perfecta en áreas menos subjetivas del instrumento.

## Introducción

Anualmente, nacen más de 15 millones de prematuros en el mundo^1^, siendo la prematuridad considerada la principal causa de admisiones en Unidad de Cuidados Intensivos Neonatales (UCIN). Además de la prematuridad y sus complicaciones, otras situaciones clínicas del período neonatal requieren cuidados intensivos.

Así, la UCIN está destinada al Recién Nacido (RN) que requiere cuidados complejos en diferentes grados y tiene como finalidad ofrecer asistencia a la salud, con recursos tecnológicos, profesionales de calidad y seguridad^2^. El enfermero neonatologista necesita identificar necesidades específicas de cada RN para la planificación y organización de la asistencia de enfermería segura y de calidad. En este sentido, algunas herramientas pueden apoyar a los enfermeros neonatologistas en la identificación de resultados de acciones en la práctica clínica^3^, en la evaluación de la complejidad de la carga de trabajo cognitiva de enfermería^4^, en la medición de la intensidad de los tratamientos y para la planificación del personal de enfermería^5^.

Con el objetivo de clasificar al RN de acuerdo con el grado de dependencia de cuidados de enfermería, en 2000 se elaboró un instrumento orientado al perfil de RN en UCIN^6^. Inicialmente, el instrumento contemplaba 16 áreas de cuidado: termorregulación, peso, actividad espontánea, reacción a estímulos, color de la piel, tonicidad muscular, nutrición e hidratación, oxigenación, integridad cutánea y mucosa, temperatura, frecuencia respiratoria, frecuencia cardíaca, presión arterial, saturación de oxígeno, control de drenajes, sondas y catéteres, y terapéutica medicamentosa.

En 2005, el instrumento fue reestructurado para el contexto brasileño, con el fin de adecuarlo a las necesidades individualizadas de cuidado, eliminando el área de nutrición e hidratación. De este modo, la versión destinada al uso en Brasil está compuesta por 15 áreas de cuidado^6^, siendo esta la versión utilizada en este estudio.

A pesar de que el instrumento de evaluación y clasificación de RN haya pasado por un proceso de validación de contenido, no se ha identificado un estudio que haya investigado su confiabilidad en la práctica clínica, lo cual es incentivado por las autoras del instrumento^6^. El análisis de confiabilidad de instrumentos implementados en la práctica asistencial es fundamental para la seguridad de la aplicación del instrumento, proponer ajustes y dimensionar los cuidados de enfermería al recién nacido^3^ ^)-(^ ^5^.

De este modo, con el fin de contribuir con el proceso de validación del instrumento, el objetivo de esta investigación fue analizar la confiabilidad de los ítems que componen el instrumento para clasificación de RN de acuerdo con el grado de dependencia de los cuidados de enfermería en una UCIN.

## Método

### Tipo del estudio

Se trata de un estudio metodológico que analizó la concordancia y la confianza de un instrumento para la clasificación del recién nacido de acuerdo con el grado de dependencia de los cuidados de enfermería en una unidad de cuidados intensivos neonatales.

### Lugar de recolección de datos

El estudio se realizó en la UCIN de un hospital privado en la ciudad de Curitiba, Paraná, Brasil. En este hospital, el instrumento para la clasificación del RN según el grado de dependencia de cuidados de enfermería^6^ se utiliza en la rutina asistencial, habiendo sido incorporado al sistema de registro electrónico del paciente en 2017.

### Período

La recolección de datos de este estudio ocurrió en el período de diciembre de 2021 a febrero de 2022.

### Población

Participaron en el estudio seis enfermeros de la UCIN y 35 RNs admitidos en el sector en el período de recolección. En la UCIN del hospital, los principales motivos para la internación de los RNs fueron la prematuridad y/o el disconfort respiratorio.

### Criterios de selección

Todos los enfermeros del sector fueron seleccionados para participar en la investigación, teniendo como criterio de inclusión ser especialista en neonatología, mientras que el criterio de exclusión fue estar de vacaciones o ausentarse durante el período de recolección de datos. Todos los RNs admitidos en el sector durante el período de recolección, cuyos padres aceptaron participar en la investigación, fueron incluidos en el estudio, sin criterio de exclusión. 

### Definición de la muestra

La muestra fue probabilística, sistemática, en la que se consideró una proporción mínima de 210 evaluaciones y máxima de 420 evaluaciones del conjunto de RNs participantes. Se realizaron 224 evaluaciones. El promedio de evaluación por RN fue de ocho días consecutivos.

### Variables del estudio

Las variables del estudio comprenden las áreas del instrumento para clasificación de RN^6^, son ellas:

- Termorregulación - capacidad de mantenimiento de la temperatura corporal estable, con gasto calórico y consumo de oxígeno mínimos, para una adaptación extrauterina exitosa;
- Peso - necesidad de control de peso para comparar el peso diario con el peso al nacer y con el peso del día anterior, ayudando en la evaluación de las condiciones nutricionales y riesgos potenciales derivados del peso de nacimiento;
- Actividad espontánea - habilidad para mantener el estado de conciencia, la respuesta comportamental a estímulos sensoriales, propioceptivos, bioquímicos, térmicos y mecánicos y parámetros fisiológicos adecuados para una adaptación extrauterina exitosa;
- Reacción a estímulos - capacidad de responder a estímulos sensoriales, propioceptivos, bioquímicos, térmicos y mecánicos adecuados para una adaptación extrauterina exitosa;
- Color de la piel - capacidad de mantener la piel y mucosas coloreadas adecuadamente para una adaptación extrauterina exitosa;
- Tonicidad - capacidad de mantenerse con tono muscular vigoroso adecuado a una adaptación extrauterina exitosa;
- Eliminaciones - habilidad para mantener eliminaciones urinaria e intestinal espontáneas con ayuda de terceros o por drenajes y estoma;
- Oxigenación - aptitud para mantener la permeabilidad de las vías aéreas y el equilibrio en los intercambios gaseosos por sí mismo o con ayuda del equipo de enfermería y/o de equipos;
- Integridad cutáneo-mucosa - capacidad de mantener la piel y mucosas sin daño o destrucción;
- Cuidado corporal - capacidad de mantener la higiene personal, vestuario;
- Control de signos vitales (SSVV) - necesidad de observación y controles de los parámetros vitales -temperatura (T), frecuencia respiratoria (FR), frecuencia cardíaca (FC), presión arterial (PA), saturación de O_2_;
- Control de sondas y drenajes - necesidad de observación y control de los equipos conteniendo fluidos de infusión y/o drenaje;
- Control de catéteres venosos - necesidad de observación y control de los catéteres de infusión y/o recolecciones, monitorización hemodinámica y nutrición parenteral hipertónica;
- Terapéutica medicamentosa - utilización de los diversos medicamentos terapéuticos - drogas, soluciones, sangre y hemoderivados;
- Educación a la salud - habilidad, confianza y seguridad de la madre/familia para dispensar cuidados adecuados para la manutención de los hábitos de salud personales y/o ambientales de los RNs.

### Instrumentos utilizados para la recolección de información

El instrumento utilizado para la evaluación y clasificación del RN^6^ está compuesto por 15 áreas de cuidado, conteniendo una pregunta para cada área y asignando un peso de uno a tres para cada ítem. El peso uno corresponde al menor grado de dependencia de los cuidados de enfermería y el peso tres, al mayor^6^. Para fines de análisis del puntaje general de dependencia de cuidados de enfermería, todas las respuestas se suman, resultando en una puntuación mínima de 15 y máxima de 45 puntos. La suma de los puntos corresponde a tres categorías de cuidado: mínimo (15 a 25 puntos), intermedio (26 a 36 puntos) e intensivo (37 a 45 puntos). Para la estandarización de los puntajes, las autoras del instrumento definieron el significado de cada categoría y asignaron la distribución del puntaje, teniendo en cuenta la inestabilidad clínica del RN y las respuestas ofrecidas por los jueces, después de aplicar la técnica Delphi, con un índice de concordancia superior al 70%^6^.

Hubo comunicación entre la autora principal del instrumento de clasificación^6^ y la primera autora del presente manuscrito para presentar el objetivo de la investigación y aclaraciones. Debido a la publicación del instrumento en su totalidad, no hubo autorización formal, solo conocimiento del uso.

### Recolección de datos

Los 35 RNs fueron evaluados diariamente, de manera doble y en el mismo momento por la enfermera investigadora y por un enfermero asistencial, con el llenado del instrumento concurrente a la evaluación. La investigadora no interfirió en la evaluación del enfermero. Estas estrategias se utilizaron para minimizar la variabilidad en la recolección de datos entre los evaluadores.

### Tratamiento y análisis de los datos

Para fines de descripción de los 15 ítems que contemplan las áreas de cuidado, se calculó la distribución de frecuencias absoluta y relativa de cada área de cuidado en la que hubo concordancia entre la enfermera investigadora y el enfermero asistencial. Lo mismo se realizó para el total de casos concordantes (n y % de concordancia).

Para la evaluación de la confiabilidad de cada ítem del instrumento, se utilizó el coeficiente Kappa ponderado (*K*p), considerando que las opciones de respuestas no eran dicotómicas^7^. El *K*p varía de 0 a 1, siendo que, cuanto más cercano a 1, mayor es el indicativo de concordancia entre los evaluadores. Para la interpretación del valor de *K*p, los intervalos correspondientes fueron clasificados de la siguiente manera: menor que 0,00 - concordancia insignificante; de 0,00 a 0,20 - concordancia débil; de 0,21 a 0,40 - concordancia razonable; de 0,41 a 0,60 - concordancia moderada; de 0,61 a 0,80 - concordancia fuerte; y de 0,81 a 1,00 - concordancia casi perfecta^8^.

Para la evaluación de la confiabilidad del puntaje general de dependencia de cuidados de RNs generado por la suma de todas las respuestas, se utilizó el Coeficiente de Correlación Intraclase (CCI). Para fines de interpretación de él, se empleó la siguiente clasificación: menor que 0,5 - baja confiabilidad; de 0,5 a 0,75 - confiabilidad moderada; de 0,75 a 0,90 - buena confiabilidad; y mayor que 0,90 - excelente confiabilidad^9^.

Por último, para la visualización de la diferencia en las medidas entre la evaluación de la enfermera investigadora y del enfermero asistencial y eventuales tendencias de esas diferencias en relación con la media del puntaje de dependencia de cuidados de RNs, se construyó el gráfico de Bland-Altman.

Todas los análisis fueron realizados con el auxilio del *software Statistical Package for the Social Sciences* (SPSS), versión 23.0, con excepción del *K*p, que fue calculado con la ayuda de la plataforma electrónica *VassarStats*. En todas los análisis, se adoptó el nivel de significancia del 5%.

### Aspectos éticos

La investigación fue aprobada por el Comité de Ética en Investigación de la Pontificia Universidad Católica del Paraná, bajo el Dictamen nº 5.127.442/2021. Las personas que accedieron a participar en el estudio firmaron el Consentimiento Informado (CI) dirigido a cada grupo: enfermeros y padres/responsables por el RN.

## Resultados

De los seis enfermeros participantes, cuatro llevaban trabajando más de cinco años en el área de neonatología y reportaron haber recibido capacitación para el uso del instrumento hace aproximadamente de uno a tres años; los otros dos enfermeros llevaban menos de cinco años en el área de neonatología y no habían recibido capacitación para la aplicación del instrumento.

La Edad Gestacional (EG) de los RNs admitidos varió de 29 a 39 semanas, teniendo un promedio de RNs evaluados con 34 semanas de EG, con una desviación estándar de tres semanas más o menos. Los diagnósticos de admisión más frecuentes fueron disconfort respiratorio, con 49% (n = 17), y prematuridad, con 23% (n = 8).

Conforme presentado en la Tabla 1, las áreas control de catéteres venosos (95%), oxigenación (93%), peso e integridad cutáneo-mucosa (92%) y control de signos vitales (90%) presentaron un porcentaje de concordancia alto (≥ 90%). Al considerar el *K*p, los ítems que presentaron concordancia fuerte o casi perfecta (*K*p > 0,6) fueron: control de catéteres venosos (0,93), oxigenación (0,84), peso (0,82), termorregulación (0,68) y cuidado corporal (0,66).

**Tabla 1:** Distribución de la frecuencia absoluta y relativa de los puntajes, del porcentaje de concordancia entre evaluadores, *K*p* e IC^†^ 95% para las áreas de cuidado del instrumento (n‡ = 224). Curitiba, PR, Brasil, 2023

Área de cuidado	1		2		3		Total		*K*p*	IC^†^ 95%
	n^‡^	%^§^	n^‡^	%^§^	n^‡^	%^§^	n^‡^	% de concordancia		
Termorregulación	92	41	6	3	61	27	159	71	0,68	0,62 - 0,75
Peso	81	36	124	55	1	0	206	92	0,82	0,75 - 0,90
Actividad espontánea	89	40	23	10	6	3	118	53	0,21	0,12 - 0,30
Reacción a estímulos	94	42	16	7	2	1	112	50	0,15	0,08 - 0,23
Color de la piel	120	54	27	12	7	3	154	69	0,45	0,55 - 0,65
Tonicidad	102	46	54	24	2	1	156	70	0,42	0,31 - 0,53
Eliminaciones	188	84	5	2	0	0	193	86	0,19	0,03 - 0,35
Oxigenación	166	74	8	4	35	16	209	93	0,84	0,76 - 0,92
Integridad cutáneo-mucosa	195	87	1	0	11	5	207	92	0,60	0,42 - 0,77
Cuidado corporal	39	17	17	8	111	50	167	75	0,66	0,56 - 0,74
Control de signos vitales	1	0	201	90	0	0	202	90	0,07	0,00 - 0,22
Control de sondas y drenajes	62	28	26	12	9	4	97	43	0,40	0,35 - 0,47
Control de catéteres venosos	126	56	8	4	78	35	212	95	0,93	0,89 - 0,97
Terapéutica medicamentosa	56	25	39	17	25	11	120	54	0,44	0,36 - 0,52
Educación en salud	112	50	20	9	8	4	140	63	0,29	0,19i - 0,39

*Kp = Coeficiente Kappa ponderado; ^†^IC = Intervalo de Confianza; ^‡^N = Número absoluto; §% = Porcentaje

En la Tabla 1, se presentan los valores del *K*p para cada una de las áreas de cuidado del instrumento. Se identificó una fuerte concordancia entre los participantes y un valor bajo de *K*p para las áreas de eliminaciones y control de signos vitales. Las áreas de cuidado peso, oxigenación y control de catéteres venosos demostraron un grado de concordancia casi perfecta. El área de cuidado reacción a estímulos reveló un grado de concordancia débil.

La media del puntaje general de dependencia de cuidados de RNs de la enfermera investigadora fue de 23,57 (±5,23), mientras que la de los enfermeros asistenciales fue de 25,19 (±6,16). El CCI entre el puntaje general de dependencia de cuidados de RNs de la enfermera investigadora y de los enfermeros asistenciales fue de 0,94, indicando una fuerte confiabilidad entre las dos medidas (Figura 1a). Conforme presentado en el gráfico de Bland-Altman, la diferencia media entre las medidas de la enfermera investigadora y de los enfermeros asistenciales fue de -1,62 (±2,69), con intervalo de confianza (95%) contenido entre 5,19 puntos. No se observó tendencia de desvío de esta diferencia de acuerdo con la media del puntaje (Figura 1b).

**Figura 1:** (a) Gráfico de dispersión de las puntuaciones para la categorización de cuidado realizadas por la investigadora y por los enfermeros asistenciales; (b) Gráfico de Bland-Altman para la diferencia y la media de las puntuaciones para la categorización de cuidado realizadas por la investigadora y por los enfermeros asistenciales. Curitiba, PR, Brasil, 2023

## Discusión

La calidad de un instrumento puede verificarse mediante el análisis del criterio de confiabilidad. Por este criterio, se observa la capacidad del instrumento para generar resultados consistentes por diferentes usuarios^10^. Otros investigadores han realizado estudios de análisis de confiabilidad de instrumentos y los resultados están contribuyendo a la calidad y seguridad en la utilización de instrumentos, impactando positivamente en la práctica asistencial^11^ ^)-(^ ^14^.

Las áreas de peso, oxigenación y control de catéteres del instrumento para clasificación de RNs según el grado de dependencia de cuidados de enfermería analizado en esta investigación tuvieron una concordancia casi perfecta, según el *K*p.

Con relación al peso, por tratarse de un parámetro totalmente objetivo, había expectativas de que la concordancia fuera perfecta entre los participantes, lo cual no ocurrió. Esta situación puede justificarse por la forma en que se presentan las descripciones de las puntuaciones del área de peso en el instrumento. El puntaje 3, por ejemplo, está indicado para puntuar al RN que tenga un peso superior a 500 g o independientemente del peso, lo que puede causar dificultades de interpretación y toma de decisiones, dado que hay necesidad de establecer límites más precisos. Para minimizar esta limitación, la descripción podría especificar que el puntaje abarca peso > 500 g y < 1.000 g, haciéndola más asertiva y dirigida. En el mismo sentido, la descripción del puntaje 2 podría cambiarse a > 1.000 g y ≤ 2.500 g.

La medición del peso en unidades neonatales es una acción frecuentemente ejecutada por el equipo de enfermería. La medición fundamenta evaluaciones importantes, como las condiciones nutricionales y la exposición a potenciales riesgos relacionados con las alteraciones del peso, que demandan organización de la dinámica de cuidados del equipo de enfermería^6^. Por lo tanto, es imprescindible un registro correcto del peso para contribuir con el puntaje que clasificará el grado de dependencia de cuidados del RN, orientando la planificación de la asistencia.

Considerando el área de cuidado de oxigenación, igualmente es imprescindible que el enfermero registre adecuadamente indicadores clínicos de alteración del patrón respiratorio del RN. Esto puede contribuir, por ejemplo, al uso racional de oxigenoterapias, previniendo posibles complicaciones^15^ ^)-(^ ^16^.

El área de oxigenación del instrumento permite establecer un valor que denota la evaluación de la permeabilidad de las vías aéreas y la homeostasis de los intercambios gaseosos, con o sin ayuda de oxigenoterapia^6^. Aunque las evaluaciones entre los participantes de la investigación mostraron una concordancia casi perfecta, las descripciones de cada puntaje pueden generar dudas en el momento de la elección. El análisis de los puntajes 2 y 3 de esta área del instrumento (2 - RNs sometidos a oxigenoterapia en la incubadora o por cánula nasal, halo o nebulización continua; 3 - RNs sometidos a halo, nebulización continua, *Continuous Positive Airway Pressure* (CPAP) nasal o ventilación pulmonar mecánica) lleva a la posibilidad de que un RN pueda estar en halo y recibir puntaje 2, pero también podría recibir el puntaje 3, dado que el término halo no está contextualizado específicamente para ninguno de los puntajes. Lo mismo ocurre con el RN que hace uso de nebulización continua.

Para adecuar la situación de repetición de atributos en puntajes diferentes, una posibilidad sería la descripción específica de las modalidades de soporte a que se refiere cada puntuación. Así, se sugiere, por ejemplo, para el puntaje 2, la descripción Cánula nasal de oxígeno, halo o nebulización continua, mientras que para el 3, podría ser CPAP o ventilación mecánica. Además, se podría explicitar la lógica de asignar la situación de puntaje 2 o 3 para cuando el RN esté en halo, lo que podría contribuir a la minimización de dudas de selección de puntaje de esta área ampliamente utilizada y evaluada en el instrumento.

De igual manera, la terapia endovenosa se aplica frecuentemente al contexto de la neonatología, demandando más tiempo del equipo de enfermería, tanto en función del número de medicamentos a ser preparados e infundidos como de la atención y cuidado a lo largo del proceso. Además de sus beneficios, puede implicar alteraciones del bienestar, dolor, complicaciones como infección y extravasamiento en la piel^17^ y angustia^18^. El enfermero debe estar atento a las evidencias científicas disponibles para potencializar efectos positivos y atenuar los negativos. 

Aunque el área del instrumento de control de catéteres venosos destacó en el porcentaje de concordancia entre los investigadores, presenta la posibilidad de diferentes interpretaciones de las alternativas relacionadas con los puntajes. Por ejemplo, si el enfermero identifica un RN con un catéter en vaso umbilical, podría dudar sobre qué puntaje seleccionar en el instrumento, ya que tanto para el puntaje 2 como para el 3 de esta área, se describe la cateterización de vasos umbilicales, sin detallar en qué situación seleccionar una puntuación u otra.

Bajo análisis detenido, se percibe que la diferencia en la descripción para los puntajes 2 y 3 consiste en las expresiones RN sometido y RN que necesita de cateterización de vasos umbilicales. Esta descripción puede guiar la interpretación a la luz de la diccionarización de tales términos. Se puede interpretar que, para el RN sometido, se puntúa el escore 2, considerando que se trata de aquel que fue sometido a la cateterización de vasos umbilicales debido a una circunstancia secundaria, como la dificultad de acceder a una vía venosa periférica.

En cambio, para el RN que necesita, se puntúa el escore 3, considerando que se cateterizaron los vasos umbilicales para un fin previamente analizado, como la incompatibilidad de la osmolaridad o pH de un medicamento con otra vía que no sea la elegida o, aún, procedimientos como exanguinotransfusión. Como las descripciones pueden inducir diferentes interpretaciones, esto puede traer dificultad para la aplicación de esa área del instrumento y, consecuentemente, la clasificación del RN.

Aunque se identifican fragilidades en el contenido de ítems del instrumento que pueden dificultar la interpretación precisa, permite que el enfermero evalúe situaciones complejas e importantes, así como las registre de forma adecuada. La evaluación y el registro denotan si el RN está respondiendo al cuidado recibido, permitiendo analizar los resultados sensibles a las acciones de enfermería, lo que determinará el establecimiento de la carga de trabajo y la planificación de la asistencia.

El RN presenta numerosas respuestas que son originadas de estímulos sensoriales, propioceptivos, bioquímicos, térmicos y mecánicos^6^, como la percepción del dolor que, aunque puede ser percibida de manera más atenta por el equipo de enfermería, tiene manifestaciones como el llanto, estando este no siempre relacionado con el dolor propiamente dicho sino con otras situaciones, como el disconfort emocional^19^.

La débil concordancia entre los participantes respecto a la reacción a estímulos puede ser explicada por la dificultad de diferenciar las descripciones de esta área de la de actividad espontánea. Se describe el área de reacción a estímulos como la “capacidad de responder a estímulos sensoriales, propioceptivos, bioquímicos, térmicos y mecánicos adecuados para una adaptación extrauterina exitosa”^6^.

Por su parte, el área de actividad espontánea se describe como la “habilidad de mantener el estado de conciencia, la respuesta comportamental a los estímulos sensoriales, propioceptivos, bioquímicos, térmicos y mecánicos y parámetros fisiológicos adecuados para una adaptación extrauterina exitosa”^6^. Se observa que los términos capacidad de responder (relacionado con la identificación de respuestas del RN) y habilidad de mantener (relacionado con la identificación de parámetros de mantenimiento del RN) pueden ser fácilmente confundidos, aunque tengan significados diferentes.

Aunque las áreas de eliminaciones y control de signos vitales presentaron un considerable porcentaje de concordancia (> 85%), por la determinación del valor de *K*p, se identificó baja confiabilidad. Esta inconsistencia se debe a la ausencia de respuestas para una de las categorías, sea por la enfermera investigadora, sea por los enfermeros asistenciales. El Kappa es un método indicado para matrices cuadradas, es decir, las dos variables a ser analizadas deben poseer el mismo número de categorías. Sin embargo, en este estudio, la pequeña variabilidad de las respuestas en estos dos ítems hizo que algunas opciones de respuesta no obtuvieran registro; por lo tanto, el presupuesto de matriz cuadrada del índice Kappa fue violado, invalidando su interpretación. De esta manera, para fines de interpretación, parece ser prudente, en estos casos, basar las conclusiones en el porcentaje de concordancia, hasta que estudios con evaluaciones más heterogéneas sean realizados.

Esta situación puede ser explicada por el hecho de que los participantes no diversificaron sus respuestas, concentrándolas en solo dos opciones. En el caso, las opciones que componen el área de eliminaciones del instrumento permiten puntuar si el RN presenta autonomía y/o disfunción del sistema digestivo y/o genitourinario. El escore 3 está designado para el RN que presenta alteraciones en los patrones de eliminaciones, con ostomías, control de las eliminaciones por peso de pañal y/o presencia de catéter vesical y/o bolsa colectora, siendo que ningún RN evaluado en esta investigación necesitó de intervenciones como cateterismo vesical o estomía de eliminación.

Esto también ocurrió con los resultados relacionados con el área de signos vitales, dado que las evaluaciones para el área requieren modificaciones expresivas para ser identificadas en el instrumento. En el contexto de la UCIN, los signos vitales son evaluados cada cuatro horas, junto con otros cuidados prestados, evitando el manejo innecesario del RN.

Aunque se identificaron variaciones en las evaluaciones de las áreas de cuidado del instrumento, esto no redujo la confiabilidad de la evaluación para la definición de la categorización del cuidado (mínimo, intermedio e intensivo) por medio de su utilización. Esto fue identificado por la comparación de la suma de las áreas de cuidado, que reveló una fuerte correlación entre los participantes de la investigación (CCI de 0,94). Sin embargo, se sugiere que la aplicación del instrumento sea acompañada por un manual, que puede minimizar la subjetividad en la interpretación del contenido del instrumento y aplicación^20^. Tal manual debe ser elaborado con explicaciones detalladas de términos y sus contextos en el instrumento, además de la certificación de la capacitación realizada.

Entre las limitaciones de la investigación se puede mencionar el hecho de que no se midieron otras propiedades psicométricas del instrumento y que se llevó a cabo durante el período de la pandemia de *Coronavirus disease*-2019 (COVID-19), lo cual debilitó las oportunidades de presencia y participación de padres y/o responsables en actividades junto a los RNs en la unidad. Esta limitación interfirió en la evaluación del área de educación a la salud, ante la necesidad de participación significativa de la familia en el ambiente neonatal para que el enfermero pueda evaluar esa área de cuidado, considerando experiencias y comprensión sobre acciones de cuidado e información compartida.

El enfermero tiene el papel primordial de actuar como facilitador junto a la familia, instrumentalizándola para los cuidados asociados a las necesidades del RN y favoreciendo la formación del vínculo afectivo entre padres y RN^21^.

La aplicación de un instrumento que evalúe el grado de dependencia de los cuidados de enfermería permite que se identifiquen necesidades de la clientela que serán relevantes para la planificación de la asistencia, así como datos sobre la carga de trabajo, lo que favorecerá el adecuado dimensionamiento del equipo de enfermería. El uso de un instrumento puede contribuir a que los enfermeros, incluso con desiguales grados de experticia, planifiquen adecuadamente la asistencia de enfermería para diferentes clientes y/o contextos. 

## Conclusión

Entre las 15 áreas del instrumento para la clasificación de RN según el grado de dependencia de los cuidados de enfermería, se verificó una concordancia casi perfecta para las áreas de cuidado de peso, oxigenación y control de catéteres venosos. La concordancia fue débil para el área de reacción a estímulos. El instrumento es confiable para clasificar a los RN según el grado de dependencia de los cuidados de enfermería en UCIN, con un CCI de 0,94.

Aunque ocurrieron variaciones en las evaluaciones de las áreas de cuidado propuestas por el instrumento, esto no redujo la confiabilidad del instrumento en la categorización del tipo de cuidado neonatal, clasificado en mínimo, intermedio e intensivo. Se sugiere que la utilización del instrumento sea acompañada por un manual que contenga la definición de términos que requieren interpretación y/o contextualización para los escenarios asistenciales.
